# Engineered Bioactive Polymeric Surfaces by Radiation Induced Graft Copolymerization: Strategies and Applications

**DOI:** 10.3390/polym13183102

**Published:** 2021-09-15

**Authors:** Mohamed Mahmoud Nasef, Bhuvanesh Gupta, Kamyar Shameli, Chetna Verma, Roshafima Rasit Ali, Teo Ming Ting

**Affiliations:** 1 Advanced Materials Research Group, Center of Hydrogen Energy, Universiti Teknologi Malaysia, Jalan Sultan Yahya Putra, Kuala Lumpur 54100, Malaysia; roshafima@utm.my; 2 Malaysia-Japan International Institute of Technology, Universiti Teknologi Malaysia, Kuala Lumpur 54100, Malaysia; Kamyar@utm.my; 3 Bioengineering Laboratory, Department of Textile Technology, Indian Institute of Technology, New Delhi 110016, India; Bhuvanesh.Gupta@textile.iitd.ac.in (B.G.); vermachetna18@gmail.com (C.V.); 4 Radiation Processing Technology Division, Malaysian Nuclear Agency, Kajang 43000, Malaysia; tmting@nuclearmalaysia.gov.my

**Keywords:** antimicrobial polymer surfaces, radiation induced graft copolymerization, protective fabrics, biomedical devices, tissue engineering materials, food packing films

## Abstract

The interest in developing antimicrobial surfaces is currently surging with the rise in global infectious disease events. Radiation-induced graft copolymerization (RIGC) is a powerful technique enabling permanent tunable and desired surface modifications imparting antimicrobial properties to polymer substrates to prevent disease transmission and provide safer biomaterials and healthcare products. This review aims to provide a broader perspective of the progress taking place in strategies for designing various antimicrobial polymeric surfaces using RIGC methods and their applications in medical devices, healthcare, textile, tissue engineering and food packing. Particularly, the use of UV, plasma, electron beam (EB) and γ-rays for biocides covalent immobilization to various polymers surfaces including nonwoven fabrics, films, nanofibers, nanocomposites, catheters, sutures, wound dressing patches and contact lenses is reviewed. The different strategies to enhance the grafted antimicrobial properties are discussed with an emphasis on the emerging approach of in-situ formation of metal nanoparticles (NPs) in radiation grafted substrates. The current applications of the polymers with antimicrobial surfaces are discussed together with their future research directions. It is expected that this review would attract attention of researchers and scientists to realize the merits of RIGC in developing timely, necessary antimicrobial materials to mitigate the fast-growing microbial activities and promote hygienic lifestyles.

## 1. Introduction

The development of various materials with antimicrobial properties has enabled the medical progress in the past several decades to take place [1]. The key function of these materials relies on their surface properties such as adhesion, biocompatibility, nontoxicity biomimicry and microbial retention/extinction [2]. Particularly, the antimicrobial properties are of utmost significance in preventing/reducing spreading of infectious diseases and combating the continuous emergence of new microbes with drug resistance. On the other hand, antimicrobial properties are not only needed to reduce the risk posed to human health by the contamination of medical tools, implants and other healthcare products by bacterial colonization forming biofilm, but also to enhance the performance of many industrial processes including food processing and packing, marine transport, and water treatment [3,4]. Despite the immense research efforts aiming at elimination of microbial proliferation and biofouling formation, a comprehensive solution to microbial surface colonization has not been reached and the quest to design and fabricate new antimicrobial surfaces remains a high research priority [5]. Thus, the development of new antimicrobial materials to eliminate/substantially reduce the extent of bacterial attachment and biofilm formation to surfaces remains of utmost significance and continues to receive intensive efforts.

Surface modification of existing polymers is one of the most effective means to impart desired functionality to polymer substrates for a wide range of applications [6]. Various surface modification methods varying from physical to chemical approaches with each approach has own pros and cons, but chemical methods are favored for efficiency and property endurance. Therefore, the selection of a particular modification method always determines the level of enhanced properties and their stability in the functionalized polymers. Chemical-induced graft copolymerization is a well-established method for surface modification of polymeric surfaces to endow covalent functionalities that has been practices for many years. However, this method is marred by its detrimental residues and the difficulty in controlling the level of grafting, and overall, it poses environmental concerns because of the use of hazardous chemical initiators and solvents [7]. Alternatively, treatment of surfaces with radiation-induced graft copolymerization (RIGC) using low-energy radiation (UV and plasma) or high-energy radiation (γ-rays or EB) as initiators leads to activation of polymer surfaces allowing reaction with specific polar monomers (under varying conditions relevant to each initiator) to yield polymers with chemically (covalently) bonded functionalities. The level of modification (grafting yield) can be easily controlled by variation of reaction parameters. The versatility of RIGC made it possible to modify almost all polymer surfaces, especially with the presence of various polar monomers allowing the creation of desired functional properties including physicochemical and biological characteristics such as antimicrobial activity and biocompatibility [8].

**Figure 1:**
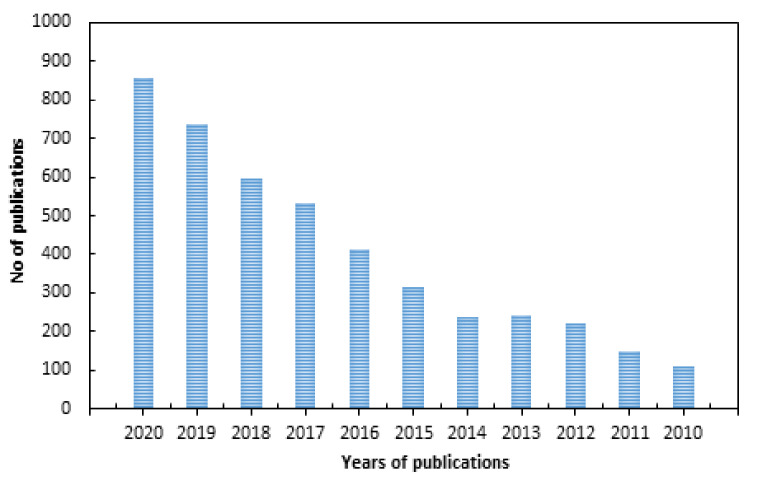
Number of publications on the topic of imparting antimicrobial properties to polymer surfaces for various applications in period of 2010–2020 (Science Direct, 22/10/2020).

There have been many research articles addressing surface modifications of polymer surfaces to impart antimicrobial properties using various physical and chemical techniques, as indicated by the large number of publications in the past 10 years shown in Figure 1 (according to a Science Direct search up to the year 2020). The classification of antimicrobial polymers and their interaction mechanisms were reviewed extensively [9,10,11,12]. Reviews with limited scope, such as those addressing individual surface modification techniques such as photo-grafting for surface modification [13], plasma treatment [14], modification of specific polymers such as silicon [15], fabrics (cotton and wool) [16], antimicrobial biomaterials containing quaternary ammonium [17], modification of biomaterials (polymer, ceramics, and metals) to impart biocompatibility and cellular interaction [18] have been published. Other articles reviewed strategies pertaining to bio-fouling prevention on silicon and silicon-based materials [15] and the use of nanometals to impart antimicrobial properties to polymers and textile fibers [19]. On the other hand, general strategies for designing antimicrobial surfaces and material selection [20] and the role of surface protection mechanisms in affecting future developments of the advanced antibacterial and antiviral materials were also reported [21]. However, a review dedicated to the use of RIGC strategies to engineer surface modifications for imparting antimicrobial properties to variety of polymeric materials of different physical forms used in developing protective fabrics, health care products, medical devices, biomedical materials, and food packaging films can be barely found in literature.

The objective of this article is to review the progress taking places in engineering of bioactive properties to various polymeric surfaces with covalently bound molecules and brushes using RIGC with different radiation sources and their applications. The scope covers different strategies used to impart antimicrobial properties to polymeric surfaces. Particularly, the advances in the use of UV, plasma, EB and γ-rays for covalent immobilization of biocides to polymers surfaces of various morphologies (e.g., fabrics, fibers, films, tubes and scaffolds) are reviewed. The emerging approaches to enhance the antimicrobial properties such as in-situ formation of metal nanoparticles (NPs) in radiation grafted substrates containing ionic groups and the use of alternative electrospun nanofibrous scaffolds are also elaborated. The current applications of grafted polymers with antimicrobial surfaces in various fields such as healthcare, biomedical, medical devices and food packaging are presented together with the challenges and future research directions to promote the use of these materials.

## 2. Polymers with Antimicrobial Properties

Antimicrobial polymers are interesting materials having potential to replace the existing biocides in various areas including medical, biomedical, health care, agricultural and packaging and textile applications [11,12]. Thus, polymers with antimicrobial properties have become a widely researched area focusing on solving various problems associated with the contamination of solid surfaces by microorganisms developing versatile and adaptive mechanisms for the colonization of surfaces and adversely affecting their functions and users [9]. Such microbial effects are most likely profound when solid surfaces are in direct contact with the human body and in the presence of high surface area combined with the ability to retain moisture in materials such as fabrics [22]. This has driven the quest of development of antimicrobial materials to prevent or reduce the formation of colonies or biofilms on materials’ surfaces and control the extent of bacterial attachment and proliferation processes [3,23,24].

Many polymers and coatings with antimicrobial properties are known to date. They can be classified into (i) biocidal polymers having inherent antimicrobial activity repelling bacterial attachment or biofilm development, (ii) polymeric biocides comprising polymer backbones with attached antimicrobial organic molecules, (iii) polymeric biocides comprising polymer backbones with attached antimicrobial inorganic compounds for contact killing and (iv) polymers with biocidal activity imparted by chemical modifications [25]. The first class of biocidal polymers showing the antimicrobial activities includes polysiloxanes, methacrylic polymers, polyoxazolines, polymers with synthetic peptides, polymers with arylamide and phenylene ethynylene backbones, polymers containing halogens, polymers containing phospho- and sulfo-derivatives, organometallic polymers, polymeric carriers with metal NPs, hyperbranched and dendritic polymers and guanidine-containing polymers. The second class of biocidal polymers is representing polymers containing antimicrobial organic compounds, which can be incorporated by a non-covalent immobilization of antimicrobial agents, either natural (e.g., immobilization of chitosan on polypropylene, PP, nonwoven fabric) or synthetic (e.g., embedding triclosan in films of nylon) on a nonactive polymer. Alternatively, this class of biocidal polymers can be obtained by blending antimicrobial polymers and non-active polymers to endow them with biocide characteristics (e.g., blending of chitosan with polyurethane or polyethylene oxide). The third class of polymeric biocides having antimicrobial inorganic compounds in which the antimicrobial activity is imparted to the polymer backbone by incorporation of inorganic compounds such as metals (e.g., AgNPs), complexed metal ions (e.g., Cu^2+^), metallic oxides (e.g., TiO_2_) and other oxides (e.g., ZnO_2_). The fourth class of polymeric biocides comprising polymer backbones with attached antimicrobial organic molecules is representing polymers with antimicrobial activities endowed by chemical modifications that can be carried out by different methods. Notably, the selection of the modification method constantly controls the structure and the properties conferred to the polymer. Different chemical modification approaches are used to achieve antimicrobial activity into polymers. They include covalent immobilization of low molecular weight antimicrobial compounds (e.g., covalently bonding cyclic N-chloramine or cyclic halamines moieties into polyester fabric/nylon-6 or PP fibers), the coupling of antimicrobial peptides to an inactive polymer (e.g., grafting of polystyrene beads surfaces with polyethylene glycol followed by loading an antimicrobial peptide) and the grafting of antimicrobial monomer onto regular polymers (e.g., graft copolymerization of poly(ethylene terephthalate) (PET) films with 4-vinylpyridene and subsequent quaternization with hexyl bromide to obtain pyridinium groups). More details on these methods together with various polymers with antimicrobial properties and the mechanisms of their bactericidal actions were published on several occasions [9,10,11,12].

## 3. Strategies for Imparting Antimicrobial Properties to Polymers

**Figure 2:**
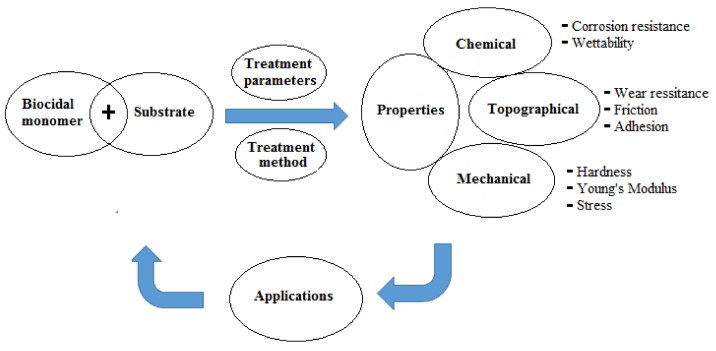
Schematic diagram for critical issues in designing antimicrobial surfaces.

The basic principles for the development of surfaces with antimicrobial properties and manipulation of microbial stability in biological environments relay mainly on (i) surface treatment parameters for the applied technique and (ii) the induced surface chemistry (e.g., corrosion resistance). The former affects the topographical properties (e.g., wear resistance, friction, and adhesion) whereas the latter modulates the wettability characteristics. Both sets of parameters ultimately control the biological/microbiological cell response on the modified surfaces in addition to their mechanical properties (e.g., hardness, Young’s modulus, and stress), which maintain the stability of the bulk of the modified substrates [24,26]. To prevent cell bacterial colonization, surface modification should induce either super-hydrophilicity (θ ≤ 10°) or super-hydrophobicity (θ ≥ 150°) [26]. A schematic diagram showing the critical properties which should be considered for designing antimicrobial surfaces is presented in Figure 2.

**Figure 3:**
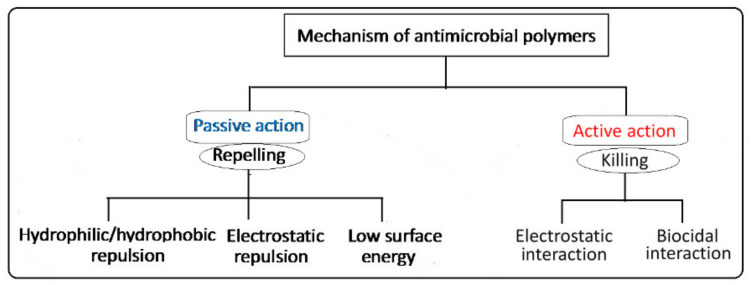
Schematic diagram of mechanism driven strategies to import antimicrobial properties and their respective mechanisms. Reprinted from [11], published by MDPI.

The design of antimicrobial surfaces has been focusing on prevention of bacterial adhesion, proliferation and biofilm formation. This can be carried out using two main strategies classified based on two action methods: (i) passive defense (bio-barrier formation) and (ii) active attack (controlled release) mechanisms [27]. The passive defense involves the direct incorporation of antimicrobial agents (e.g., polyethylene glycol, albumin, polyphenols and zwitterionic polymers) to form a barrier preventing the initial bacterial adhesion. This strategy is based on hydrophilic/hydrophobic repulsion and electrostatic repulsion, or it can be driven by low surface energy, as depicted in the schematic diagram shown in Figure 3. On the other hand, the active attack strategy involves the incorporation of synthetic biocides such as quaternary ammonium and tertiary amine working by electrostatic interactions, or natural agents (e.g., nisin and essential oils) working by biocidal interactions to kill bacteria [11,28,29]. A variety of other antimicrobial agents such as phosphonium salts, triclosan (2,4,4-hydrophenyl trichloro (II) ether) and N-halamine have been employed for inhibiting bacterial growth and fouling prevention. The mechanism of the bactericidal action of such biocides is known to involve destructive interactions with the cell wall and/or cytoplasmic membranes [30]. Nevertheless, most of these biocides are toxic or carcinogenic and pose an environmental concern when applied to preformed surfaces such as fabrics. Furthermore, the alternative natural-based biocides such as essential oils, chitosan and peptides are likely to be degradable and difficult to withstand sterilization conditions. Thus, imparting covalent immobilization of antimicrobial properties to polymeric surfaces by graft copolymerization of monomers having biocidal activities or alternatives that can be activated in a post-grafting treatment is highly effective and demanded.

Various studies have been carried out using both strategies to induce antimicrobial properties, which were proven to be effective but challenged by significant issues such as durability and loss of function by the accumulation of dead bacteria [31]. Thus, the criteria for selection of antimicrobial agents for surfaces’ treatments includes not only high efficiency against wide spectrum of microorganisms and non-toxicity to the users and environment but also substrate durability and absence of impacts on functional properties [22]. Moreover, the design of an antimicrobial surface must satisfy additional requirements such as a facile and inexpensive modification method, water insolubility, resistance to emission of toxic products and having a non-irritating nature [32].

## 4. Covalent Immobilization of Antimicrobial Properties to Polymers

Various surface modification techniques have been developed in past few decades to impart antimicrobial agents to different substrates with various morphologies such as fabrics, fibers, nanofibers, films and nanocomposites by physical and chemical methods [22]. Particularly, polymer surfaces can be modified by incorporating bioactive agents through covalent bonding, hydrogen bonding or blending of biologically active functional groups (e.g., amino, amide and carboxyl) during polymer synthesis [33].

Fabric form is among the widely used polymeric materials in many sportswear, health care and biomedical applications. Due to the nature of fabrics structures including a large surface area and the ability to retain moisture, they are more exposed to bacterial activities, causing a range of undesirable effects on both the textile itself and on the user [22]. Thus, a variety of fabric treatment methods have been developed for applying antimicrobial coatings, including pad-dry-cure, conventional exhaustion, padding, spraying, polymer grafting of active agent during polymerization, encapsulation of metal NPs by in- situ formation of some heavy metals NPs in polymer surfaces and chemical modification with active agents by binding with polymer surfaces covalently [34]. The latter method involves the use of graft copolymerization to alter properties of polymer substrate surfaces to meet specific applications.

Graft copolymerization offers advantages over other methods in terms of ability to alter wide range of properties from improving elasticity, adhesion, moisture absorption for better wicking and dyeability to thermal resistance and reactivity, with their levels of conferral being dependent upon the conditions of the grafting reactions. More interestingly, this method allows the development of polymeric surfaces having higher ionic charge densities and a greater ease of tuning the hydrophobic/hydrophilic balance for optimal polymer stability, regio-selectivity, and antimicrobial activity [9]. Moreover, surfaces modified by graft copolymerization show long-term activity due to the covalent bonding of antimicrobial agents [35].

Graft copolymerization can be initiated by several initiators as resembled from the method names, which are chemically induced grafting, photo-induced grafting, plasma-induced grafting and high energy-induced grafting with γ-rays or EB [36]. However, chemically initiated graft copolymerization, which is commonly applied as a finishing technique for fabrics (stain repellence, flame retardance, dyeing and antibacterial treatments), generally produces detrimental residues affecting product purity and resulting in a lot of polluted wastewaters [37]. Obviously, the difference in each grafting method depends on the initiation step, which varies in each method from one initiator to another. Of all methods, radiation-induced graft copolymerization (RIGC) is the most convenient method to covalently bind various forms of antimicrobial agents with desired levels in addition to its simplicity and green nature [38].

### 4.1. Radiation-Induced Graft Copolymerization

RIGC is a facile and attractive method for selectively and covalently imparting new properties originating from polar monomers into preformed polymer substrates without altering their inherent properties using a variety of radiation sources [39]. The process was invented long ago by a French scientist, Adolphe Chapiro [36], and a British scientist, Arthur Charlesby [37], but has subsequently made enormous contributions in designing and developing innovative surfaces for highly sensitive and technologically important domains. This method provides desired control over the type and level of grafted moiety and the GY is a function of grafting parameters [40]. Moreover, it helps maintain the purity of the product, which is free of detrimental residues and thus exerts lower environmental impact and provides safer eco-friendly antimicrobial surfaces [41]. Moreover, it confers an inherent sterilization caused by using high-energy radiation (γ-rays and electrons) for the treatment of substates. Thus, RIGC has been applied for the modification of polymer surfaces for the control of bacterial adhesion and growth in various occasions [42,43,44]. RIGC can be initiated in the presence of polar monomers by high-energy sources such as Co-60 and EB accelerators. Moreover, low-energy sources such as UV and plasma sources were also used to initiate surface grafting. Despite the tradition of limiting RIGP to grafting initiated with high-energy radiation such as γ-rays and electrons without any referral to radiation type, a distinctive name is given for each method to be able to compare them with graft copolymerization with low-energy radiation. Thus, four methods denoted γ ray- and electron-induced graft copolymerization are discussed parallel to photo- and plasma-induced graft copolymerization methods, which are frequently used in imparting antimicrobial properties to polymer surfaces.

#### 4.1.1. γ-ray- and Electron-Induced Graft Copolymerization

RIGP can be carried out by high-energy radiation sources such as Co-60 and EB accelerators. The former produces γ-rays, which have the advantage of deep penetration into the substrates, allowing bulk grafting depending mainly on the grafting yield (GY), which depends on the absorbed dose, dose rate and monomer concentration, but the irradiation process takes a long time, particularly when the dose rate is low. On the other hand, EB produces electrons, giving it the advantage of short irradiation time (few minutes) as well as a high and controlled dose rate, and it is more convenient for pre-irradiation methods. However, the penetration depth of EB depends on the acceleration energy of electrons and the density of the substrate [39]. Various types of accelerators include low energy (0.1–0.3 MeV), medium energy (0.5–5 MeV) and high energy (5–10 MeV) types which are available for surface curing and polymer processing [45].

When ionizing radiation interacts with polymer substrate, active species or radicals are formed through hydrogen abstraction in hydrocarbon substrates or fluorine detachment in the case of fluorocarbon polymers. Such radicals are responsible for reaction with monomer molecules in their vicinity to form macroradicals responsible for incorporating side chain grafts upon propagation and subsequent termination. RIGC with γ-rays and electrons can be generally performed with two techniques: simultaneous irradiation and pre-irradiation methods [39]. The former method involves exposing the monomer and the substrate to γ-rays at a low dose together with addition of a small amount of an inhibitor (Fe^2+^ or Cu^2+^ ions) to minimize the homopolymerization. In the latter method, the substrate is irradiated separately and subsequently exposed to the monomer under either vacuum or inert atmosphere. The irradiation step can be carried out under vacuum forming radicals or in oxygen to favor the generation of hydroperoxyl radicals. The graft copolymerization is initiated by the thermal decomposition of the formed trapped radicals in the presence of heated monomer molecules. The reaction can be carried out in a bulk neat monomer, a solvent diluted monomer or in an emulsion medium, in which a small amount of surfactant is added to a monomer solution diluted with water. The GY yield is a function of reaction parameters including monomer concentration, absorbed dose, temperature, and time. Unlike plasma- and photo-induced grafting, γ-ray- and electron-induced grafting can achieve bulk modification and has been widely investigated for the preparation of various ion exchange membranes [39] and chelating adsorbent polymers [46] and polymer electrolyte membranes for electrochemical energy systems [47]. From a practical point of view, EB is more advantageous for grafting initiation and mass production applications.

#### 4.1.2. Photo-Induced Graft Copolymerization

RIGC can be also carried out by low-energy radiation such as UV light. Photo-induced grafting with UV light is a simplified technique that allows surface modification of polymer substrates for enhanced surface properties without altering the inherent properties. When UV light falls on a polymer, active species involving radicals, cations or rarely anions are formed. Of all species, radicals are the most active species; they are involved in radical polymerization reactions that take place in the presence of a photo-initiator such as benzophenone and 1-hydroxycyclohexyl phenyl ketone. Several types of photo-initiators have been used and details of their classification can be found in Ref. 7 [13]. Grafting is commonly carried out using the immersion technique, where UV irradiation is used in the presence of vinyl monomer diluted with water or methanol. Despite the suitability of photo-initiated grafting for imparting new ionic characters to polymer substrates, it is slow, yields a low grafting level and needs a photo-initiator. Moreover, a suitable selection of wavelength is a critical factor in this method [48]. Thus, this method is more suitable only for surface modification and is more effective for improving adhesion and antimicrobial surface properties of polymers.

#### 4.1.3. Plasma-Induced Graft Copolymerization

**Figure 4:**
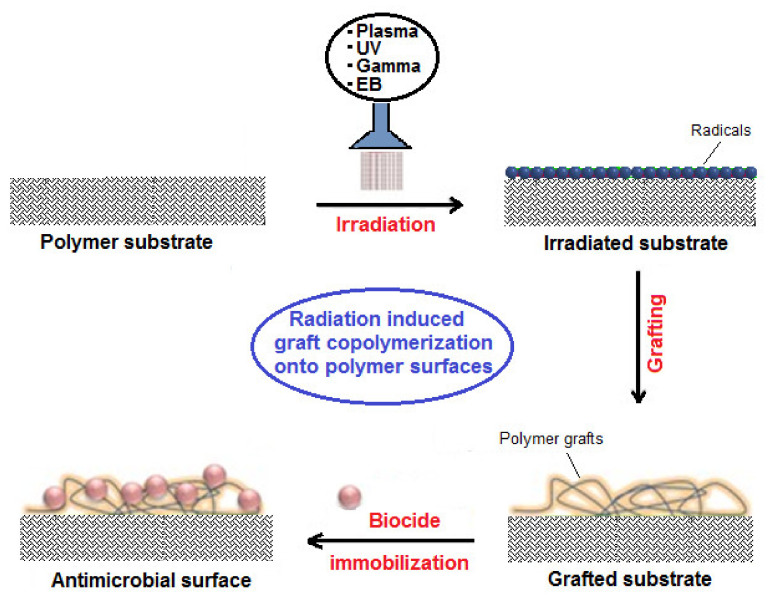
Schematic diagram of strategies for functionalization of polymer surfaces by RIGC using different radiations sources. Reprinted from [54], published by Wiley.

Plasma-induced graft copolymerization has become an attractive means for developing antimicrobial coatings using various strategies and thus, plasma-treated materials have been widely investigated [49,50]. Plasma is defined as a gaseous environment composed of mixture of active species such as free radicals, electrons, and heavy particles in addition to neutral species, making it unique and diverse media for surface modification of polymer substrates [51]. When plasma interacts with polymer surface in the presence of a monomer, both gain energy through inelastic collisions of active species and are activated. This initiates graft copolymerization, which takes place either by radicals generated on polymer surfaces followed by contact with monomer or direct grafting of polymer surfaces with activated monomers. Plasma-induced grafting requires the use of polymerizing gases such as NH_3_, N_2_, O_2_, CO_2_ and H_2_O or monomer precursors such as fluorocarbons, hydrocarbons and silicon ones where the extent of grafting depends on the plasma treatment conditions such as monomer reactivity, flow rate, system pressure, discharge power, frequency of the excitation signal and the temperature of the substrate [52]. Various plasma treatments are available and of all, low-temperature radio frequency plasma is the most frequently used technique to generate different functional groups on the material surface, depending on the nature of the gaseous medium. For example, ammonia introduces amino functionality and carbon dioxide leads to the generation of carboxyl groups, whereas H_2_O produces hydroxyl groups on polymer surfaces. The use of the plasma technique provides several advantages allowing top surface modifications with bulk damage minimization in addition of being a green technique [53]. More details on the application of plasma-induced grafting for imparting antimicrobial coatings to polymer surfaces can be found elsewhere [49]. Nevertheless, plasma- and UV-induced grafting methods are rather effective in small-scale applications in chemistry laboratories and are not practical for industrial large-scale applications [48]. Figure 4 shows a schematic diagram of strategies for the functionalization of polymer surfaces by RIGC using different radiation sources. More elaborations on the advantages and disadvantages of common RIGC methods used for modification of the polymer surfaces together with radiation sources and energetic species involved in the surface bombardment are presented in Table 1.

## 5. Designing Antimicrobial Surfaces by Radiation-Induced Graft Copolymerization

RIGC has been used for the development of polymer surfaces with antimicrobial properties and this virtually allows the use of an unlimited number of monomers for this purpose. A diverse array of vinylic and acrylic monomers and their mixtures have been copolymerized with various polymer surfaces in different physical forms to impart antimicrobial properties for several applications. The grafting of such monomers imparts electrostatic charges and companion interactions as well as hydrophilicity to the polymer surfaces, allowing modulation of bacterial adhesion and prevention of the formation of biofilm. Various monomers including quaternary ammonium compounds such as vinylbenzyltrimethylammonium chloride [55], N,N-dimethylacrylamide and [2-(methacryloyloxy)]ethyl-trimethylammonium chloride [38], N,N-dimethylaminoethyl methacrylate [56], N-vinylcaprolactam [57], acrylic acid [44], methacrylic acid [58], acrylamide [59], N-vinylpyrrolidone [60] and 1-vinyl imidazole [61] have been grafted onto various substrates for conferring antimicrobial properties. Monomers such as glycidyl methacrylate (GMA) were also grafted onto substrates such as polyethylene (PE) and propylene (PP), followed by functionalization with cyclodextrins and subsequent incorporation of the anti-fungal drug miconazole, leading to the reduction of fungal adhesion and biofilm formation in medical devices by *C. albicans*. [62].

Polymers which are suitable for imparting antimicrobial properties using RIGC are part of those used for biomedical and health care applications, which should have inherent properties such as being non-toxic, non-allergic and free of leachable products including residual monomer traces, degradation products, auxiliary products, and plasticizers. They also should have biological inertness, radiation resistance and mechanical integrity [63]. Polymers such as PE, PP, poly(vinyl chloride) (PVC), nylon, poly(dimethylsiloxane) polyurethane (PDMS-PU) and poly(vinylidene fluoride) (PVDF) are commonly used as substrates to antimicrobial acquiring treatments; however, natural polymers such as cotton fabric are also widely used.

**Figure 5:**
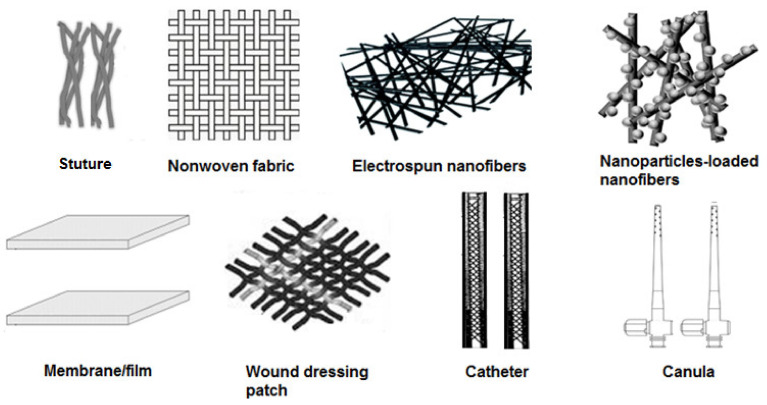
Various forms of processed polymers subjected to surface treatments to impart antimicrobial properties.

The selection of the substrates and biocidal agent for antimicrobial treatment depends on the desired applications [63]. Processed polymers/polymer composites in forms of fibers, cannulas, catheters, nonwoven fabrics, nanofibers, films, and membranes without and with NPs (schematized in Figure 5) have been subjected to surface antimicrobial treatments [34,64,65]. In this case, antimicrobial agents are coated to substrates physically (dip-coating, spray coating, spin coating, solvent casting, or layer-by-layer) or chemically/covalently (graft copolymerized) where only their surface properties are selectively changed by the imparted functionality without altering bulk properties. Particularly, chemical coatings exist in forms of crosslinked coatings, polycationic structure coatings and polymer brushes [66]. The functional properties of physically coated surfaces lack stability, unlike covalently grafted surfaces, showing better durability [67]. Therefore, RIGC has advantages to overcome low durability of the antimicrobial functions of the coated surfaces. Moreover, the use of high-energy radiation (γ-rays and EB) for grafting initiation is superior in terms of flexibility and ability to control the level of incorporated grafted functionality and practical applications.

## 6. Synergizing of Antimicrobial Properties by Metallic Nanoparticles

The progress of nanotechnology applications in medicines and biomaterials development for combating multidrug resistance in microorganisms and enhancing biocidal efficacy has been accelerated over the past 2 decades. Metallic and metal oxides NPs such as silver, copper/copper oxide and zinc/zinc oxide have been used to impart synergetic biocidal effects onto polymer surfaces and develop nano-antimicrobial biomaterials. Among metal nanoparticles, AgNPs showed a noticeable ability to kill microorganisms due to their broad spectrum of targets in cells compared to conventional antimicrobial agents. Thus, they have been directly used to impart antimicrobial activities or administered with one or more other antimicrobial agents to provide highly effective antimicrobial resistance and enhanced overall treatment efficacy [68]. AgNPs are most widely used agent in general textiles, wound dressings, and medical tools such as catheters, prosthetics, bone adhesives, contact lenses and ureteral stents. The antibacterial effects of AgNPs with respect to size and shape were addressed by Kim et al. [69].

The use of metal NPs requires careful manipulation of synthesis parameters to control their size, shape, and dispersion upon synthesis of nanostructured surfaces for antimicrobial application. Of all parameters, dispersion remains the most challenging variable to overcome the agglomeration of such NPs arising from their extremely high surface energy. Thus, careful selection of dispersion medium and surface treatment are invariably taken into consideration to ensure a uniform distribution, appropriate interactions and sufficient bonding sites for the immobilized NPs [70]. However, leaching of physically immobilized NPs poses a serious challenge, limiting the development of efficient NP-supported polymer surfaces for antimicrobial application [71]. Thus, the stability of nanoparticle-containing coatings is of particular interest and can be enhanced by synthesis of NP seeds by reduction of metal ions electrostatically bound to functional groups formed on the surface of the substrate by RIGC.

NPs can be applied in a non-supported form or in a polymer supported (nanocomposite) structure using four strategies including covalent immobilization, impregnation, sustained release of antimicrobial component and synergistic effect arising from combined action of microbial resistance from metal NPs and inherent antimicrobial action of support material [72]. NPs can be incorporated to polymer substrates by three methods: (i) ex situ nanoparticle formation and subsequent immobilization into in the surface of modified polymeric substrates, (ii) ex-situ formation of NPs and entrapping them in porous substrates and (iii) in-situ nanoparticle formation, where the polymeric substrate provides a reaction site (nanoreactor) to form NPs and a template for their subsequent immobilization [73]. Of all methods, the in-situ metal nanoparticle immobilization is the most advantageous for providing a stable association between the NPs and polymer substrates, making it the most appropriate surface modification method for imparting antimicrobial effects [72].

**Figure 6:**
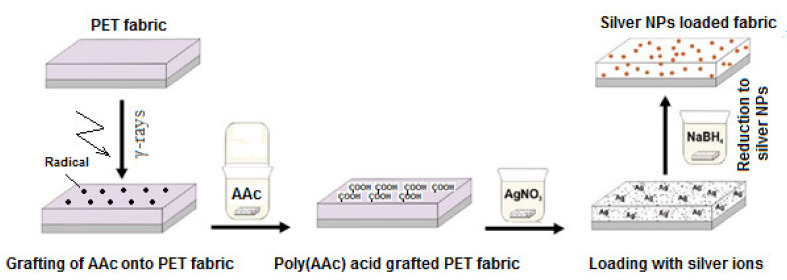
Schematic diagram of preparation of AgNP-loaded PET fabric by RIGC of acrylic acid followed by loading of silver ions and subsequent reduction to AgNPs.

Various studies report on the incorporation of stable antibacterial properties with AgNPs to several polymer substrates in the form of fibers, fabrics, and films. Poly(ethylene terephthalate) (PET), an important polymer that is used in biomedical, household and clothing applications, was endowed with antibacterial characters by in-situ incorporation of AgNPs, as reported by Ping et al. [74]. A template host was first introduced to PET films by RIGC of acrylic acid (AA) using γ-rays, followed by immobilization of silver ions and their subsequent reduction to NPs, as shown in Figure 6. The obtained surface showed high efficiency for the filtering and killing of *E. coli*, suggesting the presence of strong and stable antibacterial activity. PP in the form of sutures was also modified by RIGC using plasma irradiation of AA, followed by chitosan binding on the remaining carboxyl groups, then followed by the immobilization of silver nanoparticles, leading to sutures with suitable antimicrobial properties [75]. Another PP suture was modified by RIGC with hydroxyethyl methacrylate (HEMA) or N-isopropyl acrylamide (NIPAAm), followed by grafting of 1-vinyl imidazole (VIm) and subsequent loading of AgNPs onto the surface. The sutures with composite structures [(PP-*g*-HEMA)-g-VIm]/Ag and [(PP-*g*-NIPAAm)-g-VIm]/Ag displayed antimicrobial activity against *E coli* and *S. aureus* together with cytocompatibility. [76]. In another encounter, PU in the form of a catheter imparted a novel self-disinfection property by immobilization of zinc oxide nanoparticles (ZnONPs). The treatment was started by RIGC of a mixture of AA and GMA onto the PU catheter, followed by in-situ formation of ZnONPs. The obtained nanocomposite catheter showed excellent antibacterial and anti-biofilm formation characteristics [77]. The latest progress in development of antimicrobial surfaces with metal and metal oxides NPs coatings have been recently reviewed [78].

## 7. Applications of Grafted Polymers with Antimicrobial Properties

**Figure 7:**
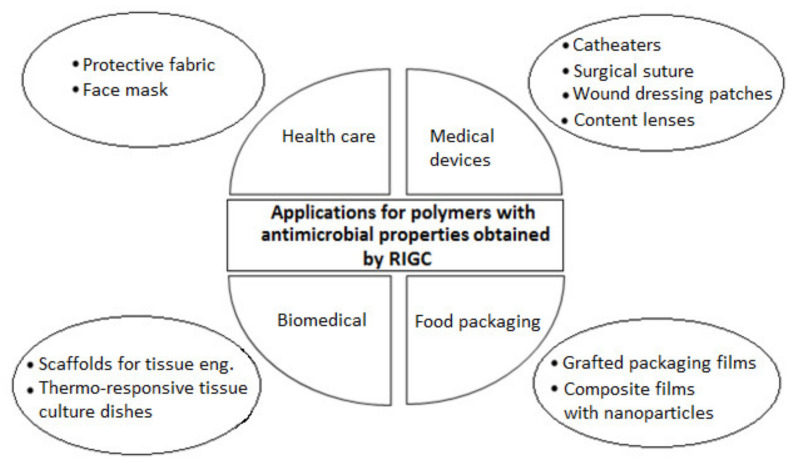
Various applications of radiation grafted polymers with antimicrobial properties.

Polymers with antimicrobial properties are receiving ever-increasing attention to mitigate the microbial resistance that is challenging medical progress and the ability to treat infectious diseases and to promote a hygienic lifestyle. Thus, polymers with antimicrobial properties prepared by RIGC have been applied in various fields such health care, medical devices, and biomedical and food packaging applications, as illustrated in Figure 7. Although the use of antimicrobial polymers in food packaging is mainly for food preserving and increasing shelf life, it is still promoting hygienic food.

### 7.1. Health Care

#### 7.1.1. Protective Fabrics

Antimicrobial fabrics are highly needed to help prevent bacterial and viral infections. Radiochemical in-situ preparation of AgNPs onto fabrics was reported on several occasions. The irradiation of silver nitrate-saturated commercial fabrics such as polyester, cotton, rayon, nylon 66, acryl, PP, and polyester/polyamide fibers with EB [79] or irradiation of fabrics such as silk in bulk aqueous silver nitrate solution without or with a stabilizer [80,81] led to the development of various Ag-loaded substrates with strong antimicrobial activities. Despite the effectiveness of this method in the formation of fabric-coated or fiber-loaded with NPs, it is challenged by the difficult control of the NPs’ size, shape and distributions. However, this topic is beyond the scope of this review and more related progress details can be found elsewhere [81,82].

Various studies reported the preparation of antimicrobial fabrics using high-energy radiation, with great interest paid to fabrics containing in-situ formed AgNPs. The AgNP-loaded cotton fibers were prepared by RIGC of GMA/iminodiacetic acid (IDA) followed by loading of Ag ions, which achieved quantities proportional to the contents of IDA, and subsequent irradiation with a UV lamp [83]. The immobilized AgNPs reached an average size of 75 nm and demonstrated high antibacterial activity against *E. coli*.

PP nonwoven fabric, a cheap and abundant melt processed polymer that have been effectively used for making a variety of disposable hygienic products such as surgical masks, diapers, wound dressings, and wipes, was used as substrate for hosting antimicrobial agents such as AgNPs to obtain the desired antimicrobial effects. Hassan and Ibrahim studied RIGC of AA onto PP fibers using γ-rays to confer antimicrobial effects followed by chelation with Ag^+^ and Zn^2+^ ions [84]. The obtained grafted fibers with polymer–metal complexes showed 100% inhibition against *S. aureus* and *E. coli*. This finding goes along with the earlier observation on antimicrobial activities of similar grafted metal complexes based on similar PP fibers obtained by RIGC [85]. Antibacterial activity against *P. aeruginosa* was also imparted to PP nonwoven fabric RIGC of AA with γ-ray and the immobilization of chitosan onto the grafted fabric through chitosan immobilized with 1-ethyl-3-(3-dimethyamino propyl) carbodiimide (EDAC) [86]. He and Gu [87] used EB to modify PET fabric to impart bacterial resistance by grafting of AA under O_2_ atmosphere using the pre-irradiation method.

A smart bio-functional PP nonwoven fabric with switchable functions from antibacterial activity to hemocompatibility was reported by Zhao et al. [88]. These authors grafted 2-(methacryboxy) ethyl)]-*N,N*-dimethylamino-ethylammonium bromide and methyl ester (CABA-1-ester) by plasma and UV treatment techniques. The grafted fabric exhibited not only significant antimicrobial activity against *S. aureus* but also outstanding hemocompatibility and antifouling properties upon hydrolysis. Spun-bond nonwoven PP fabric with antimicrobial activities against several bacteria including *K. pneumoniae* and *S. aureus* was obtained by RIGC of diallyldimethylammonium chloride (PDDA) using plasma treatment [89].

Montoya-Villegas [90] investigated the effect of graft copolymerization technique on the level of antimicrobial properties imparted to PET fabric surfaces containing AgNPs. RIGC of HEMA or a mixture of HEMA and ethylene glycol dimethacrylate (EGDMA) was grafted on PET fabric using γ-radiation and UV-radiation followed by in-situ immobilization of AgNPs. Photo-grafting yielded thinner hydrogel coating 12 times lower than its counterpart grafted using γ-radiation. The grafted fabric loaded with AgNPs equally inhibited bacterial growth against *S. aureus* and *E. coli* with higher *E. coli* inhibition in the thicker fabric [90].

Cotton fabric with antimicrobial properties was prepared by RIGC of vinylbenzyltrimethylammonium chloride (VBTC) using simultaneous irradiation with γ-rays [55]. The modified cotton fabric showed strong and stable antimicrobial activities against *S. aureus* and *E. coli* after several washing rounds. Attempts to further improve the antimicrobial properties by substituting VBTC with [2-(methacryloyloxy) ethyl]trimethylammonium chloride (MAETC) yielded cotton fabric with lesser antimicrobial properties [38]. The VBTC was also grafted in a mixture with AA of different ratios on PP sheets in comparison with their counterparts obtained by grafting with single monomers for evaluation of antimicrobial properties with a variation of degrees of grafting. The relationship between bacterial adhesion and the prepared surfaces was investigated using various tools [44]. Table 2 shows a summary of previous studies for the preparation of antimicrobial protective fabrics using RIGC.

#### 7.1.2. Face Masks

Disposable face masks, which are made from nonwoven fabrics such as cotton, polyester, or PP, are currently used as an immediate measure to protect against pathogen transmissions. However, such fabrics are far from blocking all pathogens. In fact, the disposed masks can even become a source for infections caused by microbial multiplication in their fibers. Thus, designing antimicrobial fabric having potential for application in face masks, incorporating a suitable filter material capable of not only blocking microbes but also killing and preventing their transmission, is highly sought. RIGC is an outstanding technique capable of producing fabrics with desired antimicrobial properties for such applications. Fujiwara et al. [60] studied an antimicrobial fabric made of PE immobilized with iodine developed by RIGC of N-vinyl-2-pyrrolidone (NVP) using EB followed by treatment with potassium iodide/iodine mixture and applied for making face masks [60]. The fabric displayed excellent antimicrobial activities against various microbes. The antimicrobial PP fabric was used for commercialization of a face mask by Ebara Clean Environment Co., Ltd. in response to the outbreak of the swine flu (H1N1) pandemic in 2009. The ability of iodine to be complexed with poly(NVP) prompted the use of RIGC of NVP onto PE/PP nonwoven fabric using EB to develop an adsorbent for capturing volatile iodine from fission products in nuclear power plant reactors [91].

### 7.2. Medical Devices

The interest in the development of new biomaterials with improved microbial resistance for implants and medical devices, which became essential for the health and application in several medical fields such as urology, surgery, orthopedics and dentistry, is fast growing [31]. Hence, the demand to eliminate or reduce the infections associated with polymeric implants and medical devices resulting from the adhesion of bacteria and biofilm formation causing severe and devastating complications to patients is accelerating the development of antimicrobial and antifouling devices [92,93]. Different approaches to surface design and material development have been studied in the past decade to encounter these limitations with the focus on the development of novel devices. The surface design approaches can be broadly classified into bacterial-repelling and bacteria-killing surfaces and can be covalently attached or non-covalently attached, as discussed earlier [31,94]. The common medical devices include but are not limited to catheters, cannulas, sutures, wound dressing patches and contact lenses. The materials used for medical devices include silicone, PE, PP, PVC, PMMA, polycarbonate (PC) and PU in addition to biopolymers including starch, cellulose, polyhydroxyalcanoates (PHAs) and poly(lactic acid) (PLA) [95]. PVC is the most widely used polymer for production of tools, blood bags, medical tubing, body fluids collection and enteral feeding products, whereas silicone rubber and siliconized latex are mainly used for catheters (urinary catheters) and implants in aesthetic surgeries [96].

#### 7.2.1. Catheters

Surface activation can be carried out by immobilization of bioactive molecules, either by covalent attachment surface groups or through hydrophobic/ionic interactions. The former method can be achieved by RIGC, which is highly effective in forcing stable binding of bioactive agents with common hydrophobic polymers through ionic groups [54]. RIGC using γ-rays has been used for modification of medical devices and the progress in the research on medical devices made of silicone rubber was recently reviewed [97].

**Figure 8:**
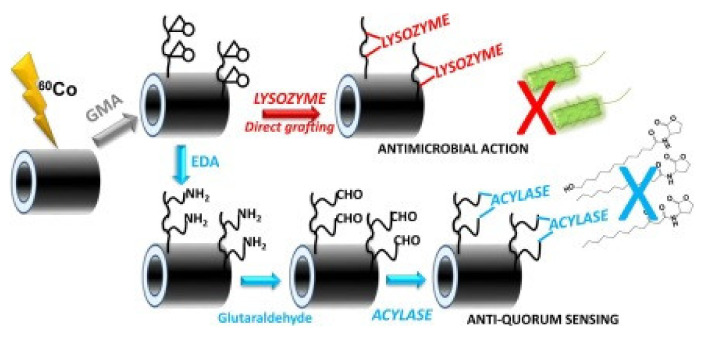
Schematic diagram for modification of PVC catheters by RIGC with GMA followed by either loading with lysozyme or treatment of ethylenediamine and glutaraldehyde acrylic acid to load acylase. Reprinted from [96], with permission from Elsevier.

Catheters made of PVC were modified by Zuñiga-Zamorano et al. [98] using RIGC of methacrylic acid (MAA) by the pre-irradiation method with γ-rays to impart a pH-responsive character. The grafted catheters with 68.5% GY displayed a 20% size increase, but more importantly, they showed an enhanced antimicrobial capability by reaction with benzalkonium chloride and ciprofloxacin, conferring them a pH stimuli response to release such antimicrobial agents. The modified catheters showed antimicrobial activity against *E. coli*, *P. aeruginosa* and *S. aureus*, and a potential to prevent/reduce urinary tract infections. Becerra et al. [96], in another study, modified the surface of PVC catheter by RIGC with GMA using pre-irradiation with γ-rays followed by immobilization of lysozyme and acylase under mild conditions, allowing preservation of antimicrobial and anti-quorum sensing performances. Lysozyme was immobilized first on the catheters hindering the adhesion of *S. aureus*, followed by covalent acylase immobilization, which was carried out by reaction with ethylenediamine and glutaraldehyde, as shown in Figure 8. The modified catheters showed anti-quorum sensing and strong potential antifouling property [96].

1-VIm was used to impart antimicrobial behavior to PVC catheters using simultaneous irradiation from γ-rays, followed by quaternization and treatment with methyl iodide, as reported in literature [99]. The microbial resistance to activities of *S. aureus* was prompted. The same monomer was used to modify silicone rubber by RIGC using simultaneous irradiation by means of γ-rays to confer antimicrobial properties. The modified silicone rubber exhibited an antibacterial activity against *P. aeruginosa* [61]. Valencia-Mora et al. [100] investigated thermo-responsive properties of silicon rubber with lower critical solution temperature (LCST) in the range of 32–34 °C by RIGC of N-vinylcaprolactam (NVCL) (water-soluble, nontoxic, thermo-sensitive and biocompatible) monomer with γ-rays using the simultaneous irradiation method and subsequent lysozyme loading. The presence of PNVCL imparted large wettability changes to temperature differences, whereas the latter introduced antimicrobial properties [100]. When the grafting reaction was carried out in toluene as a solvent, a bulk grafting was achieved. Conversely, conducting the reaction in a monomer diluted in water yielded a surface grafting. The presence of PNVCL allowed reversible load and release of lysozyme. A summary of previous studies applying RIGC for covalent immobilization of antimicrobial agents for catheter application is presented in Table 3.

#### 7.2.2. Surgical Suture

Surgical sutures are one of the most widely used medical devices for closing wounds after injury or surgery [101]. The nature of sutures’ application leaves them exposed to microbial adhesion and biofilm formation, causing wound infections. Sutures are made of natural (silk) or synthetic polymeric (PP and PET) materials with different physicochemical properties controlling their behavior upon application. Thus, it is highly important to develop sutures with not only surface retardance to microbial adherence, colonization, and biofilm formation, but also with simultaneous wound healing and drug delivery capabilities. Efforts have been undertaken to develop new sutures by incorporation of biocides to sutures with methods including blending, surface functionalization with covalent immobilization and surface coating [102]. Of all, covalent immobilization of antimicrobial or anti-inflammatory drugs by RIGC is a highly effective method for improving sutures’ antimicrobial properties to control surgery site infections [103].

Filaments/fibers of PP and PET, the most widely used materials in development of sutures, mainly due to their optimum tensile strength and biocompatibility and other interesting properties, were modified by RIGC using different monomers on many occasions [103,104]. Particularly, PP sutures modified by RIGC of monomers such as MAA [105], HEMA [106] and acrylonitrile (AN) [107] were earlier investigated using simultaneous and pre-irradiation methods with γ-rays. On the other hand, the resistance of sutures made of medical grade black braided silk and pure mulberry silk twisted yarn against bacterial infections was improved by RIGC of MAA with pre-irradiation using γ-rays followed by immobilization of 8-hydroxy quinoline hydrochloride on the grafted sutures [58].

**Figure 9:**
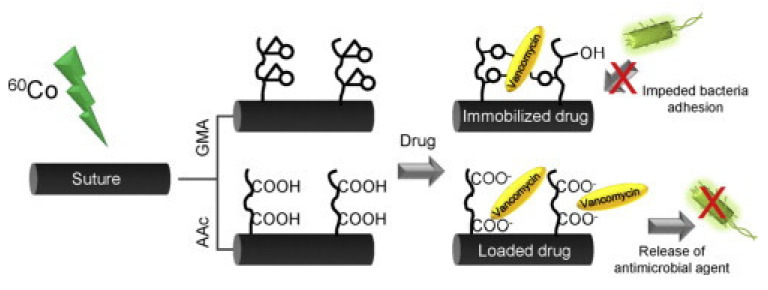
Schematic diagram for modification of PP suture by RIGC with GMA and acrylic acid followed by vancomycin loading. Reprinted from [110], with permission from Elsevier.

In another study, a PP suture (monofilament) was modified by RIGC of 1-VIm using simultaneous irradiation with γ-rays and showed much-needed antimicrobial activity when loaded with ciprofloxacin [108]. PP fibers were also modified RIGC of lauryl acrylate (LA) and butyl acrylate (BA) using simultaneous irradiation with γ-rays for potential application as sutures [109]. RIGC of GMA or AA was used to modify PP monofilament sutures by pre-irradiation method using γ-rays as reported by García-Vargas et al. [110]. The grafted suture was loaded with vancomycin using the covalent immobilization on a PGMA ligand or ionic interaction with grafted PAA moiety, as illustrated in Figure 9. The immobilization of vancomycin inhibited the activity of *S. aureus* adhered to the surface of the suture combined with pH-dependent swelling, allowing the preparation of an antimicrobial drug suture [110].

Further development involving a PP monofilament suture with thermo-responsive or pH-sensitive swelling properties was reported by López-Saucedo et al. [111]. The PP suture was modified by RIGC of N-isopropylacrylamide (NIPAAm), HEMA or 1-VIm using the pre-irradiation method with γ-rays. The suture modified with PNIPAAm displayed thermo-responsiveness and the counterpart modified with PVIm showed pH-sensitivity in addition to hydrophilicity and cytocompatibility. The authors extended their work to confer PP monofilament sutures combined thermo-responsive and pH-sensitive swelling properties with antimicrobial activity against *E. coli* and *S. aureus* by consecutive RIGC of HEMA or NIPAAm, followed by grafting 1-VIm using γ-rays and subsequent treatment with methyl iodide. The sutures grafted with the combination of PNIPAAm and PVIm showed pH and temperature responsiveness [112]. Based on previous studies, it is imperative to mention that γ-rays are highly effective for introducing an engineered bioactivity to PP sutures. Nevertheless, there is a challenge to the use of RIGC with γ-irradiation on PP filaments pertaining to the control of GY so that it takes place on the surface and avoid bulk. This is to ensure that grafting remains confined to the amorphous regions of the polymer fibers to avoid adverse impacts on the mechanical properties. This can be ensured by optimization of grafting parameters [105].

Graft copolymerization with plasma offers an effective alternative surface modification method for sutures’ applications. A CO_2_ plasma was used to introduce a carboxylic acid group to PET sutures followed by loading with bioactive Ag nanogels and chlorhexidine. The nano silver nanogel was obtained by the reduction of a silver complexed suture, which was prepared using polyethylene glycol that also stabilized the formed AgNPs (10–50 nm). The coated sutures displayed excellent antibacterial activity against both *E. coli* and *S. aureus* [104]. Similarly, CO_2_ plasma-activated PET sutures containing carboxylic group and loaded with triclosan exhibited an excellent antibacterial activity against both *E. coli* and *S. aureus* [113]. In the latest study, Angum et al. [114] introduced a bioactivity to PET sutures with carboxylic group functionalization using plasma and subsequent loading bioactive agents of Ag and its combination with Aloe vera. This imparted excellent bacteriostatic and bactericidal properties to the sutures against both *E. coli* and *S. aureus.* A summary of previous studies applying RIGC for covalent immobilization of antimicrobial agents for sutures application is presented in Table 4. More details on the progress taking place in the development of a new generation of sutures with high antimicrobial activities and drug release were reviewed elsewhere [103].

#### 7.2.3. Wound Dressing Hydrogels/Patches

**Figure 10:**
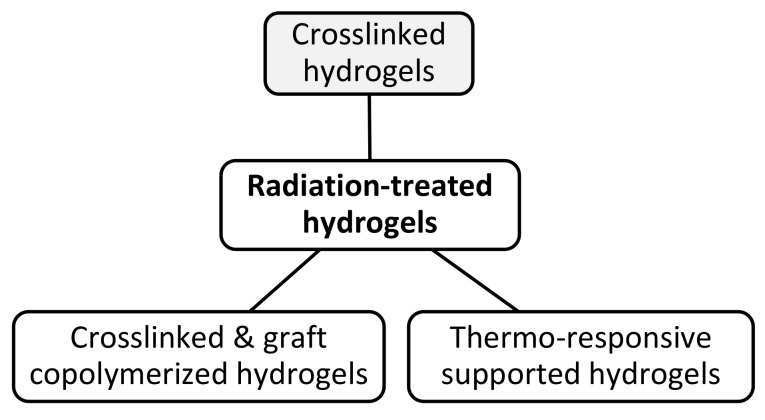
Schematic diagram of radiated processed hydrogel for wound dressing.

A wound dressing material is a sheet ideally providing an optimal healing environment by maintaining a moist medium at the wound surface, allowing gas exchange, acting as a barrier to microorganisms, removing excess exudates but not reaching saturation on the wound’s outer surface, stopping the wound desiccation, reducing the wound surface necrosis, stimulating the growth factor, providing mechanical protection to the wound, and having comfortable removal [115,116,117]. In addition, such materials should be non-allergic, non-toxic, biocompatible, biodegradable, easily sterilizable and commercially acceptable [118]. Polymeric materials applied for wound dressings are used in different forms, including sheets, foams, hydrogels, alginates, and hydrocolloids. Natural synthetic polymers and their combinations have been also used for preparing dressings and the majority exist in crosslinked hydrogel forms [119]. The preparation of wound dressing hydrogels is carried by chemical and irradiation methods. However, a small number of products were developed by the irradiation methods with majority of these products belonging to synthetic wound dressings. The preparation of hydrogel wound dressings involves three steps: formation of initial mixture solution (e.g., polyvinyl pyrrolidone, PVP and polyvinyl alcohol (PVA)), shaping into sheet in sealed plastic packages and finally, irradiation with the sterilization dose. Such hydrogels can act as smart polymers capable of slow drug release where drugs can be administered through the hydrogel in-situ. Radiation-processed hydrogels prepared by high-energy radiation such as γ-rays and EB can be categorized into three classes, including crosslinked hydrogel, crosslinked and graft copolymerized hydrogels and thermo-response supported hydrogels, as can be seen in Figure 10.

The use of high-energy radiation has been reported for the preparation of crosslinked hydrogels such as PVA/chitosan hydrogel membranes crosslinked with γ-rays, which showed a good resistance against *E. coli*, especially with high chitosan contents in the hydrogel membranes [120]. Other hydrogels of PVA/gelatin were also reported [121]. The presence of PVA chains improved the blood compatibility whereas chitosan imparted antimicrobial activity, which is a function of chitosan content to these membranes [122]. Furthermore, the healing capability of these PVA/chitosan hydrogel films was enhanced by the incorporation of glycerol [123] and minocycline [124]. Hydrogel films of PVA with carboxyethyl chitosan films crosslinked with EB also demonstrated very good antimicrobial activities against *E. Coli*. [125]. Another hydrogel film comprised of PVA, PVP, kappa-carrageenan and silk powder that was prepared by crosslinking with γ-rays or EB showed strong potential for wound dressing after demonstrating not only antimicrobial activity and non-toxicity but also fast wound and burn healing capabilities [126]. Hydrogels prepared by radiation-induced cross-linking PVA and gelatin and graft copolymerization of MAA [127,128] onto gelatin to produce PVA/MAA/gelatin copolymers was also reported wound dressing applications. The wide applications of hydrogel films for wound dressing signify the role that high-energy radiation can play in imparting antimicrobial activities to wound dressing polymers, although their role does not involve copolymerization but is limited to crosslinking, which is beyond the scope of this review.

Conventional wound (from burning or scratching) dressing material is composed mainly of gauze, which sticks to the newly grown tissues, causing damage to them, accompanied by pain upon wound dressing and thus, the recovery is also prolonged. To overcome this problem, supported hydrogels in a form of dressing material that can be easily stripped off the wound, giving comfort and healing support, were developed by RIGC of acrylate monomers onto nonwoven fabrics such as PET, nylon and PP. Particularly, NIPAAm monomer was grafted onto the nonwoven fabric by RIGC, initiated by γ-ray irradiation. The LCST in thermo-responsive NIPAAm was retained after the grafting and this makes the dressing fabric able to be stripped off easily without hurting the healing tissue [129].

**Figure 11:**
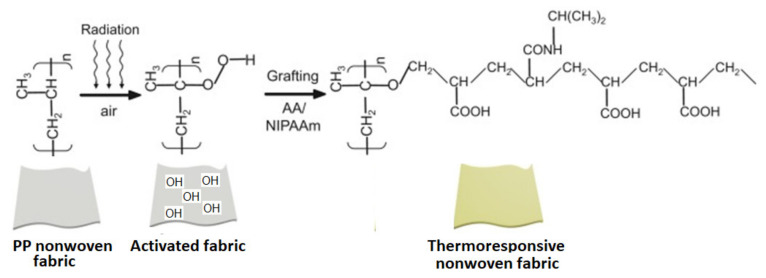
Schematic diagram for preparation of thermo-responsive supported hydrogel by RIGC of NIPAAm/AA onto PP monofilament mesh. Reprinted from [131] with permission from Elsevier.

Another version of wound dressing fabric containing chitosan having antisepsis, hydrotaxis and temperature-sensitivity properties was also developed based on similar nonwoven fabric grafted with a mixture of NIPAAm and AA, then coated with a chitosan layer and sprayed of AgNO_3_ solution [130]. A similar grafting mixture (NIPAAm and AA) was also grafted onto PP nonwoven fabric to develop a thermosensitive supported hydrogel [131]. NIPAAm and N,N′-methylene bis(acrylamide) (MBA) monomers grafted onto commercial monofilament mesh of PP were also investigated with O_2_-plasma activation, as represented by the schematic diagram shown in Figure 11. The supported hydrogel showed thermo-responsive properties imparted by incorporation of PNIPAAm, stable below and above the LCST of 33.2 °C [132].

Electrospun nanofibrous sheets were also used as alternative substrates for various biomedical applications including wound dressing patches, scaffolds for tissue engineering and drug release materials after modification with desired functionalities using RIGC. This is due to their advantages of high surface area to volume ratio, mechanical strength, 3D structures and ability to be immobilized with functional groups. A revision on grafting of macromolecules on nanofibrous surfaces using various methods and their applications in the controlled release of drugs to promote wound healing was recently published in the literature [133]. Electrospun polymer nanofibers incorporating antimicrobial agents were produced with antimicrobial capability against a wide range of microorganisms. For example, electrospun nylon-6 nanofibers were modified by UV-induced grafting (photo-grafting) of 2-substituted vinylimidazoles to impart antimicrobial activities, which were tested against *E. coli* and *S. aureus*. The obtained grafted nylon 6 nanofibers exhibited almost a complete growth reduction of both bacteria [133]. RIGC of 4-vinylpyridine (4-VP) with UV was earlier used to modify PU followed by quaternization of the grafted pyridine groups with hexyl bromide to confer antimicrobial activity. The obtained poly(4-vinyl-N-hexylpyridinium bromide) moiety grafted to the surfaces was proven to be highly effective against *S. aureus* and *E. coli* activities [[133]. More details on the application of nanofibrous substrates modified by various methods for wound dressing application can be found elsewhere [134].

#### 7.2.4. Contact Lenses

Contact lenses are one of the medical devices that are popularly used for correction of refractive errors and maintaining ocular applications such as drug depots for sustained release and assistance in healing persistent corneal defects and post-surgical corneal wounds. Contact lenses are subjected to the formation of biofilms resulting from adhesion and growth of microorganisms, causing irritation to the eyes and potential visual disability [135]. Therefore, it is highly important to develop contact lenses with antimicrobial and antifouling properties to meet their rapidly evolving demands. Several studies reported the use of graft polymerization with UV for developing potential materials with antifouling properties for contact lens applications. A potential contact lens material comprising silicone hydrogels grafted with poly(ethylene glycol) methyl ether acrylate (PEGMA) was prepared using UV-induced graft polymerization. The obtained PEGMA-grafted silicone hydrogel showed a good combination of transparency with oxygen permeability and maintained good tensile strength and elastic modulus in addition to a combination of excellent surface hydrophilicity and antimicrobial property [136]. In another study, the hydrophilic and antifouling of silicone hydrogels was improved by grafting of 2-methacryloyloxyethyl phosphorylcholine (MPC) using graft polymerization with UV. The obtained PMPC grafted silicone hydrogel possessed a combination of excellent surface antifouling character, hydrophilicity, oxygen permeability and mechanical properties [137].

Hydrophilic monomers such as HEMA and NVP were copolymerized using free radical UV-initiation in the presence of Azo-bisisobutyronitrile (AIBN) as a photo-initiator, diethylaminoethyl methacrylate (DEMA) or ethyleneglycol dimethacrylate (EGDMA) as a crosslinker to develop materials for contact lenses. The lenses fabricated from the obtained copolymer demonstrated geometrical uniformity, optical clarity, and handling ability on par with commercial lenses [138].

Plasma initiation was also used to modify contact lenses to improve their antifouling properties. Silicone lenses were modified by covalent deposition of allylamine using plasma initiation followed by grafting of poly(ethylene oxide) dialdehyde (PEO (ALD)2) [139]. A similar procedure was applied to impart antimicrobial activity against *P aeruginosa* and *S. aureus* using AA to attach a melamine-derived peptide (Mel4) to the surfaces of several silicone lenses [140]. In another study, melamine was covalently bound to silicone contact lenses, which displayed improved anti-microbial properties [141]. Various types of polymers for making contact lenses for different applications and strategies to endow them with antifouling properties were recently reviewed [135,142]. It can be concluded that grafting of bioactive agents for modification of contact lenses was mostly performed with low-energy radiation such as UV and plasma and no work could be found using high-energy radiation sources for such an application.

### 7.3. Biomedical Applications

#### 7.3.1. Substrates/Scaffolds for Tissue Engineering

The interest in designing substrates and scaffolds to support cell adhesion, growth and detachment for regenerative medicine and tissue engineering has been growing for the past few decades. Natural polymers have been found suitable for implants and not suitable for tissue engineering, unlike synthetic polymers such as polyglycolide (PGA), polylactide (PLA), poly(lactide-*co*-glycolide) (PLGA), poly(ethylene-glycol) (PEG) and poly(ε-caprolactone) (PCL). These polymers possess desired properties such as biocompatibility, biodegradability, mechanical integrity with self-reinforcement in addition to ease of manufacturing [143,144,145]. However, these polymers require surface modification not only to overcome their hydrophobicity and lack of recognizable biochemical binding sites but also to enhance cell–surface interactions, proliferation, and differentiation. Thus, modifying surfaces of such polymers by covalent grafting allows the development of various tissues for regenerative medicine.

Monomers imparting hydrophilic characters such as AA, MAA, maleic anhydride (MAN) or acrylamide (AAm) [146] can be introduced to scaffolds made of PLA and PCL using RIGC initiated by UV [147,148] and γ-rays [146,149]. Plasma activation in the presence of gases such O_2_ or NH_3_ provides another route for imparting desired properties to polyester surfaces [150,151]. The obtained substrates/scaffolds showed a strong potential for cell culture and pH-sensitivity applications. Reviews on various surface modifications of biodegradable polyester substrates (films and nanofibers) with RIGC and plasma treatment for tissue engineering applications can be found in few recently published reviews [143,144,145,152].

#### 7.3.2. Thermo-Responsive Culture Dish

Scaffolds are available in various forms including porous and fibrous hydrogel scaffolds. Biodegradable synthetic polyesters are often used as porous scaffolds, which provide cell infiltration, proliferation, and differentiation. Due to their advantages, porous and fibrous scaffolds are widely used in tissue engineering, yet they are challenged by the firm occupation of the proliferated cell with extracellular matrix depositions, leading to difficulty in the detachment of proliferated cell tissues. To overcome these limitations, a novel scaffold-free tissue engineering approach was developed by Ikano and coworkers using smart temperature-responsive cell culture surfaces to fabricate tissue-like grafts with many kinds of cells [153,154,155,156]. The new approach not only allowed non-invasive control of cell attachment, detachment, and direct transplantation of harvested cells to the host tissue without any suture, carrier, or support, but also enabled preservation of the intact cellular functions and high cell densities.

**Figure 12:**
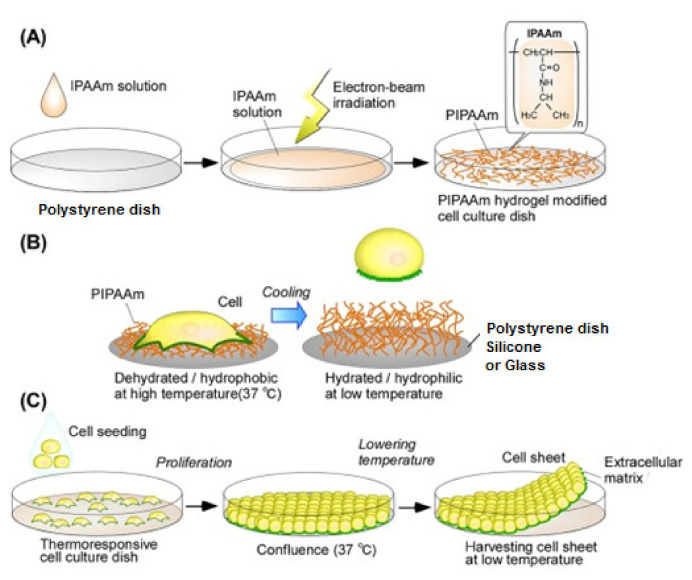
Thermo-responsive cell culture dish for thermally modulated cell adhesion, detachment, and cell sheet fabrication: (**A**) preparation of thermo-responsive cell culture dish by electron-induced graft polymerization, (**B**) temperature-dependent cell adhesion and detachment and (**C**) cell sheet fabrication using thermo-responsive cell culture dish. Reprinted from [159], published by Elsevier.

The temperature response culture surface can be obtained by incorporating PNIPAAm, a thermo-responsive polymer, on the surface of tissue culture dish (e.g., polystyrene dish), having a LCST of 32 °C, above which hydrophobic character prevails and below which it is inverted to a hydrophilic property. Therefore, variation of temperature from 37 °C to 20 °C triggers switchable hydrophobicity/hydrophilicity and tunable hydrogel properties, allowing cultured cells to be harvested as intact cell sheets [153,155,157]. A schematic diagram illustrating this approach is shown in Figure 12, whereas studies reported the preparation of various thermo-responsive culture surfaces with RIGC are listed in Table 5. A review on the principles of cell sheet engineering with temperature-responsive cell culture surfaces and their applications in regenerative medicine was published in literature [158].

PNIPAAm-grafted surfaces can be obtained by RIGC with UV [170] and EB [165,167]. However, EB irradiation is the most widely and the first used method to graft cell culture substrates with PNIPAAm [153,171]. The use of RIGC allows modification of various substrates other than polyester, including PET, silicon rubber and glass. Hydrogel scaffolds with controllable pore size, fully degradable and thermo-responsive properties were prepared by reaction of NIPAAm with 2-methylene-1,3-dioxepane (MDO) and caprolactone dimethacrylate (CLDMA) using UV copolymerization [166]. The PNIPAAm-based hydrogel scaffolds were found to be attractive for applications in tissue engineering. Another temperature-responsive cell culture surface based on poly(dimethylsiloxane) (PDMS) was investigated by Fukumori et al. [168]. The PDMS was subjected to an initial activation with silanol groups with conventional O_2_ plasma imparting more favorable hydrophilicity than treatment with HCl, which imparted hydrophobicity. The silane-loaded PDMS surface was grafted with NIPAAm using electrons irradiation graft polymerization by EB, imparting a temperature responsiveness to cell surface. Notably, the obtained smart surface has a potential application in cell culture surfaces with stretchable temperature-responsiveness. Details of the important attributes of previous studies on the use of RIGC to prepare thermo-responsive culture surfaces are summarized in Table 5. The various methods for preparation of PNIPAAm-grafted surfaces, challenges and future research directions in biomedical applications were reviewed in a few previous encounters [157,159,172,173].

### 7.4. Food Packaging Polymers with Antibacterial Properties

The interest in the development of polymers for food packaging has received ever-increasing interest to maintain food’s inherent quality during long-term storage and extend shelf life. This can be achieved using antimicrobial packaging materials, which is a typical way to suppress the activities of microorganisms during storage. Thus, an immense amount of work has been carried out to develop polymeric materials for active packaging capable of resisting bacterial activities by incorporating antimicrobial agents of different types [174]. Antimicrobial agents can be introduced to packaging polymers physically by blending, or chemically by covalent bonding, which can be carried out by RIGC where parent substrates retain their inherent properties while acquiring new hybrid functionalities through the incoming monomers. The desired properties of polymers films used for packaging should include colorlessness, flexibility, transparency, mechanical integrity, stability under low and high temperatures, resistance to oil and fats, tight gas barrier properties, sealing capability and low cost, as described elsewhere [175].

#### 7.4.1. Packaging Films with Grafted Biocidal Monomers

Various processed polymer films such as PVC, PET, polystyrene, LDPE, HDPE, and PP have been used for hosting antimicrobial agents for food packaging applications [176,177]. Among all, PP films are popular because of their inherent properties such as high thermal resistance, chemical inertness, and mechanical stability in addition to abundance and low cost. However, the strong hydrophobicity of PP poses challenges to bonding with other surfaces and thus, it must undergo surface treatments including RIGC with polar monomers to improve its surface properties. PP film was modified with monomers such as AA [178,179] and their mixture [180] using RIGC (pre-irradiation) method with EB irradiation for imparting antimicrobial properties. In another study, antimicrobial properties were imparted to PP film by pre-irradiation grafting of AA, N,N-dimethylacrylamide (DMA) and the mixtures of both AA and DMA with [2-methacryloyloxy)ethyl] trimethylammonium chloride using EB. The surface hydrophilicity varied depending on the monomer and comonomer grafted [181]. Adhesion tests for samples obtained from RIGC of N,N-dimethylacrylamide (DMA) and [2-methacryloyloxy)ethyl] trimethylammonium chloride (PMETAC) confirmed that adhesion depends mainly on the hydrophilicity and the roughness for the designed plastic surfaces for antimicrobial applications [179].

On the other hand, metal chelators with enhanced antimicrobial activity of lysozyme were acquired into PP films to obtain active packaging film by imparting an antimicrobial activity of lysozyme against *L. monocytogenes*. The modification of PP film was started by RIGC of AA using photo-initiation followed by electrostatic interactions with desired metal source. The obtained film proved the effectiveness of potential of metal-chelating active packaging films in enhancing the antimicrobial activity of lysozyme [182]. In another study, a new antimicrobial film made of ethylene vinyl alcohol copolymers (an oxygen barrier material) with covalently attached lysozymes showed antibacterial activities against *L. monocytogenes*. The film was treated with UV irradiation to generate carboxylic acid groups followed by lysozyme immobilization [183].

Modification of LDPE to acquire antimicrobial properties was also studied using different strategies. For example, a non-migrating antimicrobial active film was developed by RIGC of sorbic acid on low LDPE was reported for developing packaging film to reduce food spoilage and improve food safety [184]. Development of biodegradable packaging based on biopolymers modified with RIGC was also investigated, but to lesser extent. For example, methylcellulose showed interesting packing properties after modification by RIGC of HEMA using γ-rays [185]. More details can be found in the recent review on various materials and trends in antimicrobial food packaging by Huang et al. [177]

#### 7.4.2. Packaging Films with In-Situ Immobilized Silver Nanoparticles

The use of AgNPs was applied to impart antimicrobial properties to PET film grafted with AA using RIGC with γ-rays and subsequent neutralization with an aqueous ammonia, followed by complexation of the carboxylate anions with silver ions from the silver nitrate solution. The immobilized silver ions were reduced to AgNPs and their amount could be controlled by variation of GY in the modified film. The composite film containing sliver nanoparticles demonstrated excellent bactericidal activity. [74]

RIGC of AA with γ-ray irradiation onto PLA films and subsequent immobilization of AgNPs onto the grafted PAA was also investigated [186]. The obtained composite PLA films exhibited excellent hydrophilicity and antibacterial activity compared to the pristine PLA even at low content of AgNPs. A similar PLA composite film containing AgNPs was preparing by RIGC of NVP onto PLA film with γ-ray irradiation, followed by immobilization of AgNPs. The grafted PVP allowed the formation of a bridge to connect the AgNPs with the PLA film and their stabilization against agglomeration [187].

## 8. Challenges and Future Directions

RIGC is an old process but is making inroads into the chemical design of polymeric materials for a vast number of applications. The interesting part is that the process is applicable to almost all the polymer–monomer combinations, which is otherwise a very difficult task in chemical initiation approaches. The most interesting aspect of the process over chemical ones is that the substrates may be functionalized across the bulk irrespective of their shape and size. This could be realized by the ability of RIGC to modify surfaces of various physical forms including nonwoven fabrics, filaments, nanofibrous scaffolds, nanocomposites catheters, contact lenses and cell culture dish. Such substrates need careful designing in a way that allows the sought bioactivity to be efficiently imparted. Thus, the proper selection of monomer(s) and antimicrobial agent(s) are key components to incur specific features to such substrates using RIGC.

Radiation grafting leads to materials with very high purity, as the initiator (except in grafting with UV) is not used during the polymerization and the radiation itself acts as the initiator for the grafting. These are likely the features of radiation processing that made this process so interesting for biomaterials and other technologically interesting domains. One must look for further advancements in this direction. One of the aspects is this regard is to pursue the development of the many researched surfaces with engineered bioactivity made by RIGC from the laboratory scale to commercial products. Drug delivery and antimicrobial implants have been in use for several decades. However, more attention should be given to growing materials such as nanogels and nanocomposites, which can be designed by radiation processing with high-energy radiation and may be used for hydrogels in tissue contacting materials for human organ reconstruction. The biodegradable gel may be a better approach for radiation processing. It seems that RIGC will remain the subject of immense interest in the future as well due to its novel nature in functionalizing polymeric materials.

## 9. Conclusions

In this review, it has been shown that RIGC is an appealing and feasible method to impart durable bioactive functionalities to polymer surfaces regardless of polymer shape and size. The method allows engineering polymeric surfaces with inherently antifouling and antimicrobial properties, which are highly needed to control the ever-growing microbial infections and promote healthy lifestyles. The use of high-energy radiation (γ-rays and EB) for grafting initiation is superior to low-energy radiation (UV and plasma) initiation in terms of speed, flexibility, the ability to control the level of incorporated grafted functionality and its industrial-scale applications. RIGC allows facile endowment of stable antimicrobial properties to fabrics, which can be utilized in applications promoting hygiene and health care such as protective clothes and face masks. Moreover, this method enables the preparation of thermo-responsive properties and pH-sensitive sutures without compromising their mechanical properties and cytocompatibility. The effectiveness of RIGC in imparting antimicrobial activities to catheters and wound dressing polymers is also realized but achievements in the latter application is mostly limited to crosslinking by irradiation with γ-rays and EB. Grafting of bioactive agents for modification of contact lenses is mostly performed with low-energy radiation sources such as UV and plasma. RIGP with EB irradiation was found to be the most widely applied method to graft cell culture substrates with PNIPAAm, allowing the development of thermo-responsive cell culture dish for thermally modulated initial cell adhesion and detachment for tissue engineering.

## Figures and Tables

**Table 1:** Summary of advantages and disadvantages of common radiation-induced graft copolymerization methods and their sources.

Graft Copolymerization Methods	Treatment Source	Energetic Species Bombarding Surfaces	Advantages	Disadvantages	Remarks
EB-induced graft copolymerization	EB	Highly directionalelectrons, of variable energy	Simple and very fast. Allows surface and bulk grafting depending on acceleration energy. Leaves no detrimental residues. Can be initiated with EBs with wide range of energies.	High cost of infrastructure for irradiation Grafted materials are likely to sustain mechanical damage when high doses and dose rates are used	More convenience for practical applications and is more suitable for up-scaling and development of semi-continuous lines for industrial applications.
γ-Ray-induced graft copolymerization	Co-60	Energetic photons	Simple but slower than EB, Allows bulk grafting depending on absorbed dose and dose rate. Widely applied and most suitable for simultaneous grafting in bulk solution.	Grafting takes longer time than EB. The Co-60 source continues to decay and thus the dose rate reduces steadily, requiring adjustment of reaction parameters	Green grafting reactions can be conducted in emulsion media to significantly reduce monomer consumption and absorbed dose and improve the process economy.
Photo-induced graft copolymerization	UV	Energetic photons of lower energy compared to those of γ-rays	Simple, inexpensive and can easily modify polymer surfaces.	It yields a low grafting level, which is confined to the surface, takes long treatment time and requires the use of a photo-initiator. Not suitable for large-scale applications.	More suitable for surface modification that can help improve wettability and resistance to bacterial colonization and biofilm formation.
Plasma-induced graft copolymerization	DC glow discharge plasma	Energetic species including atoms molecules ions, radicals, photons, electrons	Simple process without any pollution to modify the polymeric surfaces without altering their bulk properties, allowing functionalization with moieties for hosting biocides.	The range of chemical groups available for surface modification is limited, posing a challenge to effectiveness for deterring bacterial adhesion. Not suitable for large-scale applications.	More suitable for biomedical application and suitable for limited surface modification such as catheters and cannulas in addition to bio-medical coatings to various surfaces.

**Table 2:** Summary of previous studies applying RIGC for covalent immobilization of antimicrobial agents for protective fabric applications.

Substrate	Monomer	Method	Bioactive Agent	Impact	Refs
PP	AA	RIGC with γ-rays	Ag, Zn and Cu metal complexes	Anti-microbial activity against *S. aureus* and *E. coli*	[85]
cotton	GMA	RIGC with γ-rays	Ag nanoparticle loaded on iminodiacetic acid	Anti-microbial activity against *E. coli*	[83]
PP	AA	RIGC with γ-rays	Chelated Ag^+^ and Zn^2+^ions	Anti-microbial activity against *S. aureus* and *E. coli*	[84]
PP nonwoven fabric	AA	RIGC with γ-rays	Chitosan immobilized with1-ethyl-3-(3-dimethyamino propyl) carbidiimide	Anti-microbial activity against *P. aeruginosa*	[86]
PP nonwoven fabric	CABA-1-ester	Plasma and UV treatments	Poly(CABA-1-ester)	Anti-microbial activity against *S. aureus*	[88]
Spun-bond nonwoven PP	PDDA	Plasma treatment	Poly(diallyldimethylamm-onium chloride)	Anti-microbial activity against *K. pneumoniae* and *S. aureus*	[89]
PET fabric	HEMA or HEMA/EGDMA	RIGC with γ-rays and UV	In-situ AgNPs	Anti-microbial activity against *S. aureus* and *E. coli*	[90]
Cotton fabric	VBTC	RIGC with γ-rays (simultaneous irradiation)	Poly(VBTC)	Anti-microbial activity against *S. aureus* and *E. coli*	[55]
Cotton fabric	MAETC	RIGC with γ-rays (simultaneous irradiation)	Poly(MAETC)	Anti-microbial activity against following bacteria sequence: S. aureus < *E. coli* < *B. cereus* < *P. fluorescens*	[38]
PP sheets	VBTC/AA	RIGC with EB (pre-irradiation)	Poly(VBTC/AA)	Anti-microbial activity against *Listeria monocytogenes*	[44]
PE nonwoven fabric	NVP	RIGC with EB pre-irradiation method	Poly(NVP)/I_2_/KI	Anti-microbial activity against *S. aureus, E. coli, P. aeruginosa*	[60]

**Table 3:** Summary of previous studies applying RIGC for covalent immobilization of antimicrobial agents for catheter application.

Monomer	Substrate	Method	Bioactive Agent	Impact	Other Functions	Refs
MAA	PVC	RIGC with pre-irradiation from γ-rays	Benzalkonium chloride and ciprofloxacin	Anti-microbial activity against *E. coli, P. aeruginosa* and *S. aureus*	pH stimuli responsive character	[98]
GMA	PVC	RIGC with pre-irradiation from γ-rays	lysozyme and acylase	Anti-fouling against *S. aureus*	anti-quorum sensing character	[96]
1-VIm	PVC	RIGC with simultaneous irradiation from γ-rays	Methyl iodide	Anti-microbial activity against *S. aureus*	-	[99]
1-VIm	Silicone rubber	RIGC with simultaneous irradiation from γ-rays	Poly(1-vinylimidazole)	Antimicrobial activity against *P. aeruginosa*	-	[61]
NVCL	Silicone rubber	RIGC with simultaneous irradiation from γ-rays	Poly(NVCL)/lysozyme	Antimicrobial activity	Thermo stimuli responsive character leading to release of lysozyme	[100]

**Table 4:** Summary of previous studies applying RIGC for covalent immobilization of bioactive functionalities for sutures application.

Monomer	Substrate	Method	Bioactive Agent	Impact	Refs
MAA	PP fibers	RIGC with γ-rays with simultaneous irradiation method	-	-	[105]
MAA	Black braided silk and pure mulberry silk twisted yarn	RIGC with γ-rays with simultaneous irradiation method	8-Hydroxy quinoline hydrochloride	Antimicrobial activity against various Gram-positive and Gram-negative bacteria	[58]
HEMA	PP monofilament	RIGC with with simultaneous irradiation from γ-rays	8-Hydroxy quinoline	Antimicrobial activity against *S. aureus*	[106]
AN	PP fibers	RIGC pre-irradiation method using γ-rays	-	-	[107]
VIm	PP monofilament	RIGC with simultaneous irradiation from γ-rays	Ciprofloxacin	Excellent antimicrobial activity against both *E. coli*	[108]
LA and BA	PP fibre	RIGC with simultaneous irradiation from γ-rays	-	-	[109]
GMA or AAc	PP monofilament	RIGC pre-irradiation method using γ-rays	Vancomycin	Antimicrobial activity against *S. aureus*	[110]
HEMA or NIPAAm followed by VIm	PP monofilament	RIGC with γ-rays using pre-irradiation method	Methyl iodide	Antimicrobial activity against *S. aureus*	[112]
-	PET sutures	Plasma treatment with CO_2_	In-situ immobilized silver nanoparticle	Excellent antimicrobial activity against both *E. coli* and *S. aureus*	[104]
-	PET sutures	Plasma with CO_2_	Triclosan	Excellent antimicrobial activity against both *E. coli* and *S. aureus*	[113]
-	PET sutures	Plasma with CO_2_	In-situ immobilized silver nanoparticle with Alvera	Excellent antimicrobial activity against both *E. coli* and *S. aureus*	[114]

**Table 5:** Summary of previous studies using RIGC for preparation of thermo-responsive culture surfaces.

Monomer	Substrate	Method	Bioactive Agent	Graft Form	Refs
NIPAAm	Polystyrene Dish	EB irradiation	PNIPAAm	Thin gel on substrate allowing successive subculturing of bovine hepatocytes	[153]
NIPAAm/AA	Polystyrene film	UV irradiation	P(NIPAAm/AA)/azidoaniline	Hydrophilic/antimicrobial/thermo-response surface	[160]
NIPAAm/AA	Polystyrene film	Glow-discharged	P(NIPAAm/AA)/insulin	Hydrophilic/antimicrobial/thermo-response surface conjugated with insulin	[161]
P(NIPAAm-*co*-CCMS)	Tissue culture polystyrene surfaces	UV-irradiation-induced crosslinking	P(NIPAAm-*co*-CCMS)/bovine endothelium or human retinal pigmented epithelium	Hydrophilic/hydrophobic antimicrobial/thermo-response surface dish	[162]
NIPAAm	Polystyrene dish	RIGC with EB irradiation	PNIPAAm	Thin gel on substrate allowing control of hydrophilic/hydrophobic property alterations and cell adhesion/detachment behavior	[163]
NIPAAm	Substrate	RIGC with γ-rays	PNIPAAm	Thin gel on substrate allowing control of hydrophilic/hydrophobic property alterations and cell adhesion/detachment behavior	[164]
NIPAAm	Glass coverslips	RIGC with EB irradiation	PNIPAAm	Thin gel on substrate allowing control of hydrophilicity and allowing temperature-dependent cell adhesion/detachment when the grafted density was around 2mg/cm^2^	[165]
NIPAAm with MDO CLDMA	-	Coplymerization with UV	P(NIPAAm/MDO/CLDMA)	Hydrogel scaffolds with controllable pore size, fully degradable and thermo-responsive properties	[166]
PNIPAAm solution	Thin film of P(NIPAAm)	Irradiation-induced crosslinking	-	-	[167]
NIPAAm	PDMS	Initial activation with silanol groups with conventional O_2_ plasma or hydrochloric acid (HCl) followed by RIGC of NIPPAmwith EB irradiation	PNIPAAm/PDMS	Gel on substrate with temperature-responsive cell culture surface	[168]
NIPAAm	Polystyrene dish	UV with photo-initiator	PNIPAAm	Hydrophilic/thermo-response surface dish	[169]

## Data Availability

Data is contained within the article.
